# Endoscopic eradication therapy with multifocal cryoballoon ablation for Barrett esophagus-related neoplasia: a prospective European multicenter study

**DOI:** 10.1055/a-2710-6551

**Published:** 2025-11-19

**Authors:** Charlotte N. Frederiks, Anouk Overwater, Torsten Beyna, Horst Neuhaus, Raf Bisschops, Roos E. Pouw, Jacques J. G. H. M. Bergman, Maximilien Barret, Roberta Maselli, Vinay Sehgal, Rehan J. Haidry, Bas L.A.M. Weusten

**Affiliations:** 1 Department of Gastroenterology and Hepatology, University Medical Center Utrecht, Utrecht University, Utrecht, The Netherlands; 2 Department of Gastroenterology and Hepatology, St. Antonius Hospital, Nieuwegein, The Netherlands; 3 Department of Internal Medicine and Gastroenterology, Evangelical Hospital Dusseldorf, Düsseldorf, Germany; 4 Department of Gastroenterology and Hepatology, University Hospital Gasthuisberg Leuven, KU Leuven, Leuven, Belgium; 5 Translational Research Center for Gastrointestinal Disorders, KU Leuven, Leuven, Belgium; 6 Department of Gastroenterology and Hepatology, Amsterdam University Medical Centers, Amsterdam, The Netherlands; 7 Amsterdam Gastroenterology Endocrinology Metabolism, Cancer Center Amsterdam, Amsterdam, The Netherlands; 8 Department of Gastroenterology and Digestive Oncology, Cochin Hospital, Paris, France; 9 Endoscopy Unit, Humanitas Research Hospital, Milan, Italy; 10 Department of Gastroenterology and Hepatology, University College London Hospitals, London, United Kingdom of Great Britain and Northern Ireland; 11 Department of Gastroenterology and Hepatology, Cleveland Clinic London, London, United Kingdom of Great Britain and Northern Ireland

## Abstract

**Background:**

Focal cryoballoon ablation (FCBA) is a relatively new modality for treatment of Barrett esophagus (BE)-related neoplasia. This study evaluated the efficacy and safety of FCBA for BE.

**Methods:**

Patients with BE segments (Prague classification C≤2M≤5) with dysplasia or early cancer were eligible for inclusion. Following endoscopic resection of visible lesions, FCBA was performed at 3-month intervals until complete eradication of BE (maximum five sessions). After ≥2 FCBA sessions, add-on treatment was allowed. Follow-up endoscopy was scheduled at 6 months and annually thereafter. Outcomes were complete eradication of endoscopically visible BE (CE-BE), intestinal metaplasia (CE-IM), and dysplasia (CE-D), durability of treatment response, and adverse events.

**Results:**

107 patients (mean age 65 years, 91 males, median BE C0M2) were included. Endoscopic resection was performed at entry in 65% (69/107). Patients received a median of 2 FCBA treatments. Add-on treatment was performed in 40% (43/107), mainly APC for small remaining islands (38%; 41/107). CE-BE and CE-D were achieved in 94% (101/107; 95%CI 90%–98%) and CE-IM in 91% (97/107; 95%CI 85%–95%), per intention-to-treat analysis. In per-protocol analysis, CE-BE and CE-D was achieved in 100% (101/101; 95%CI 100%–100%), and CE-IM in 96% (97/101; 95%CI 92%–99%). After a median follow-up of 18 months, 96% (97/101; 95%CI 92%–99%) remained free of endoscopically visible BE. Esophageal stricture was the most common adverse event, in 13% (13/101; 95%CI 6%–20%).

**Conclusion:**

FCBA was highly effective in selected patients with BE of limited length, although the potential risk for stricture formation warrants further research.

## Graphical Abstract

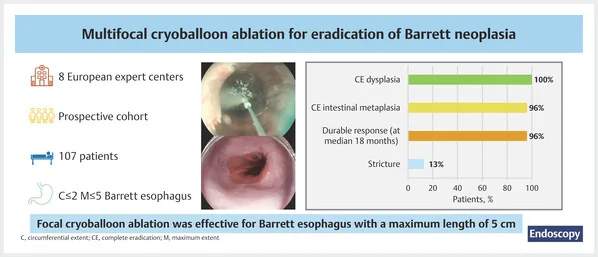

## Introduction

Barrett esophagus (BE) is an important precursor lesion for esophageal adenocarcinoma, which is believed to progress through a stepwise manner from intestinal metaplasia (IM) to low grade dysplasia (LGD), high grade dysplasia (HGD), and eventually cancer 1 . Current guidelines unanimously recommend endoscopic eradication therapy for BE with early neoplasia (i.e. LGD, HGD, or early T1 cancer). Endoscopic eradication therapy typically consists of a two-step approach involving endoscopic resection (ER), if a visible lesion is present, followed by ablation therapy for any residual flat Barrett epithelium 2 3 .

Although radiofrequency ablation (RFA) is currently the most established ablation modality in this treatment approach 4 5 6 , new endoscopic ablation techniques have been developed to overcome the limitations of RFA, including a substantial stricture rate varying from 7% to 15% 4 5 6 7 , considerable post-procedural pain and dysphagia 8 , limited availability of a device that can be applied through the working channel of an endoscope, and relatively high costs. One of these techniques is cryoballoon ablation 9 , which is associated with better patient tolerability compared with RFA 10 . In addition, cryoballoon ablation is believed to preserve the extracellular matrix, which potentially enables deeper tissue ablation 11 .

Initially, focal cryoballoon ablation (FCBA) was introduced to target small surfaces, with each ablation covering approximately 2 cm ^2^ . Previous studies have demonstrated that FCBA is a feasible, safe, and effective treatment for flat-type BE of small extent, with complete eradication rates reported to be 72%–100% 12 13 14 15 . However, these clinical trials were limited by small sample sizes, relatively short follow-up, single treatment sessions, and/or the use of the first-generation device. The aim of this prospective, European, multicenter study was to investigate whether the efficacy, safety, and tolerability of FCBA for treatment of a BE segment of up to 5 cm could be confirmed in a large, international setting.

## Methods

### Study setting

In this prospective, multicenter, intervention study, patients were included at eight European centers with a tertiary referral function for endoscopic management of early Barrett neoplasia. These referral centers all met the requirements of a Barrett expert center according to the recently revised European Society of Gastrointestinal Endoscopy guideline 3 . To ensure standardization of the FCBA technique, the principal investigator(s) of each center received hands-on training. In addition, a minimum of five lead-in cases under supervision on site by the principal investigator of the study (B.L.A.M.W.) was required prior to enrolling patients. A study representative from the coordinating center (C.N.F., A.O.) attended all FCBA procedures on site to ensure protocol adherence and high-quality data.

### Patient selection

Patients were eligible for study participation if they were aged ≥18 years and had a BE segment of limited extent (circumferential extent [C] ≤2 cm and maximum extent [M] ≤5 cm according to the Prague classification) and an indication for ablation therapy. Ablation therapy was indicated for patients with flat BE with either LGD (i.e. histopathologically confirmed during two consecutive endoscopies) or HGD in the 6 months prior to inclusion, or residual BE after ER (i.e. endoscopic mucosal resection [EMR] or endoscopic submucosal dissection [ESD]) of a visible lesion at least 6 weeks prior to inclusion containing any degree of dysplasia or low-risk early esophageal adenocarcinoma (i.e. invasion depth of <500 µm, good-to-moderate differentiation, absence of lymphovascular invasion, and tumor-negative vertical resection margins). The maximum extent of ER was limited to 2 cm in length and 50% of the circumference. Exclusion criteria were prior endoscopic ablation therapy, esophageal stricture preventing passage of a therapeutic endoscope, active esophagitis (higher than Los Angeles grade A), and esophageal varices (see **Table 1s** in the online-only Supplementary Material).

### Focal cryoballoon ablation system

**Fig. 1:**
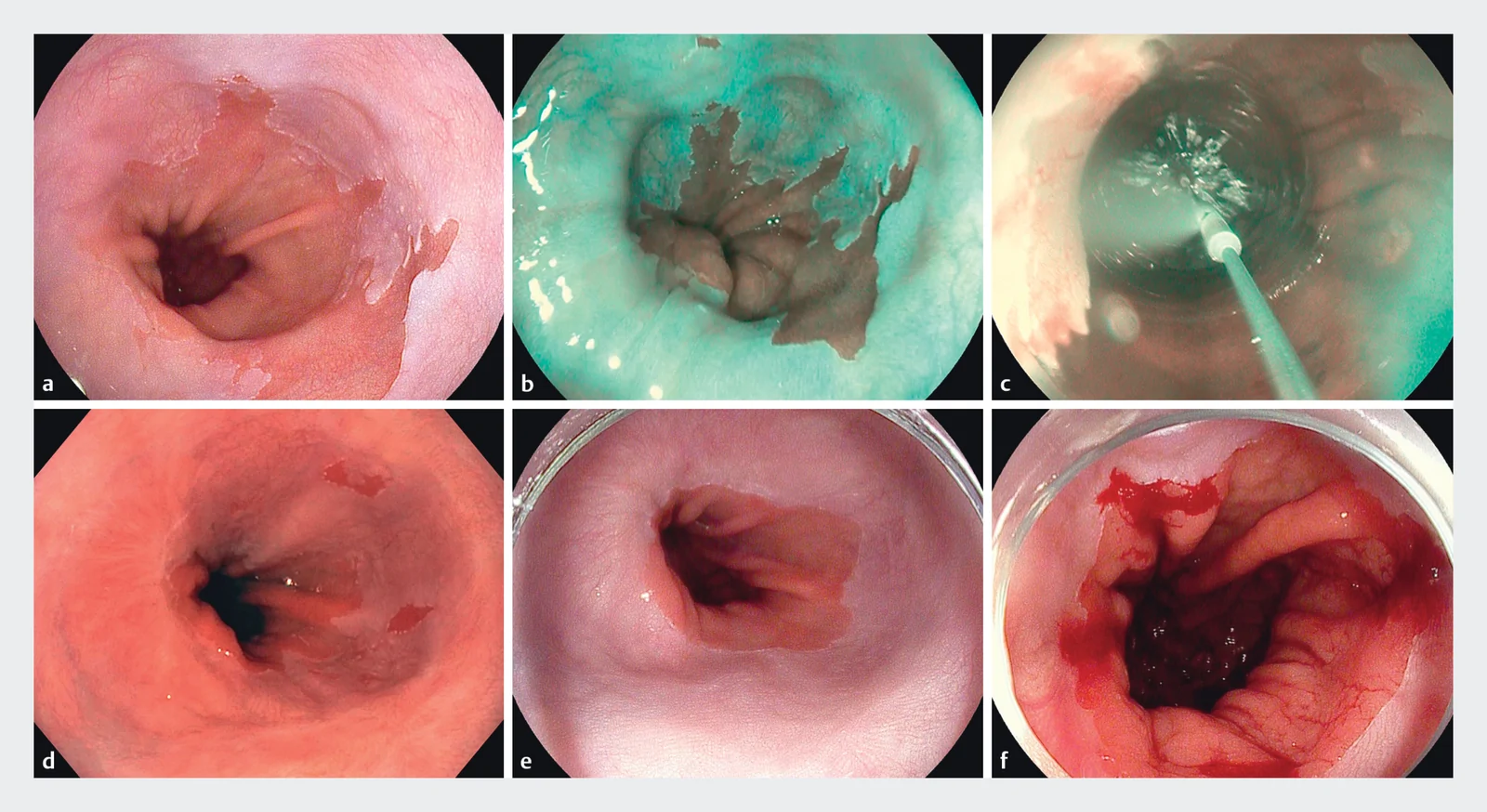
Endoscopic eradication therapy with multifocal cryoballoon ablation. a,b Endoscopic images of a flat-type C0M2 Barrett esophagus with low grade dysplasia. c During the first focal cryoballoon ablation session, the entire Barrett segment, including the gastroesophageal junction (GEJ), was treated with seven ablations of 8 seconds each. d The next endoscopy at 12 weeks showed some small remaining Barrett islands at the 1, 3, and 4 o’clock positions, which were treated consecutively along with the GEJ. e After two focal cryoballoon ablation sessions, all endoscopically visible Barrett esophagus was eradicated. f Biopsies were taken just below the GEJ, confirming complete eradication of intestinal metaplasia.

The C2 Cryoballoon Ablation system (PENTAX Medical, Redwood City, California, USA) has been described in detail previously 16 17 . In short, the next-generation system comprises a handheld controller, foot pedal, balloon catheter with rotatable spray diffuser, and disposable cartridges containing liquid nitrous oxide ( **Fig. 1s** ). For this study, we used the focal cryoballoon catheter covering a surface area of approximately 2 cm ^2^ per ablation. This catheter was available in either cylinder or pear shape at the discretion of the treating endoscopist.

### Treatment protocol

Focal cryoballoon ablation treatment.Video 1

All study procedures were performed on an in- or outpatient basis according to the site’s standard of care for anesthesia and sedation. The endoscopy system included high-resolution white-light endoscopy and i-SCAN Optical Enhancement imaging (PENTAX Medical). During each procedure, the BE segment was carefully inspected with documentation of the C&M classification. Thereafter, FCBA was performed using side-by-side ablations as described previously 16 .

Although the study originally started with a dose of 10 seconds per ablation, this initial dose resulted in a higher stricture rate than expected based on previous studies. After a temporary pause, the study was amended and restarted using 8 seconds per ablation as the standard dose 16 . This main article describes the results of the complete target population treated with 8 seconds (n = 107); details of the results from the separate, distinct cohort of patients treated with the initial dose of 10 seconds before the study was restarted (n = 28) are provided in the **Supplementary Text 1s** . The standard dose of 8 seconds could be lowered to 6 seconds at the discretion of the treating endoscopist in cases of severe scarring or to prevent high dosing in a small untreated area with risk of overlapping ablations.

The protocol dictated that the gastroesophageal junction (GEJ) at the upper end of the gastric folds was treated circumferentially during every FCBA session, followed by all visible BE including islands ( Video 1 ). FCBA treatment was performed at 3-month intervals until endoscopic clearance of BE was achieved, with a maximum of five treatment sessions ( Fig. 1 ). After at least two FCBA sessions, circumferential ablation of the GEJ could be omitted at the discretion of the treating endoscopist (e.g. significant scarring at the GEJ with high stricture risk), and add-on treatment for residual BE was allowed. Add-on treatment was performed at the same 3-month intervals immediately following FCBA, with either a single EMR for areas >5 mm or a maximum of two argon plasma coagulation (APC) sessions for diminutive residual islands of <5 mm. Any new visible lesion detected at a scheduled therapeutic session was treated with ER at the discretion of the local treating physician. When all endoscopically visible BE was eradicated, random four-quadrant biopsies were taken immediately distal to the GEJ (<5 mm) and from every 2 cm throughout the original length of the BE segment. If these biopsies showed persistent IM, one final additional FCBA of the GEJ was allowed.

During the entire treatment phase, patients received high-dose proton pump inhibitors (equivalent to esomeprazole 40 mg twice daily). Additional acid-inhibiting medication (e.g. H _2_ -receptor antagonist and/or sucralfate suspension) was prescribed after each therapeutic endoscopy at the discretion of the treating physician.

### Follow-up protocol

**Fig. 2:**
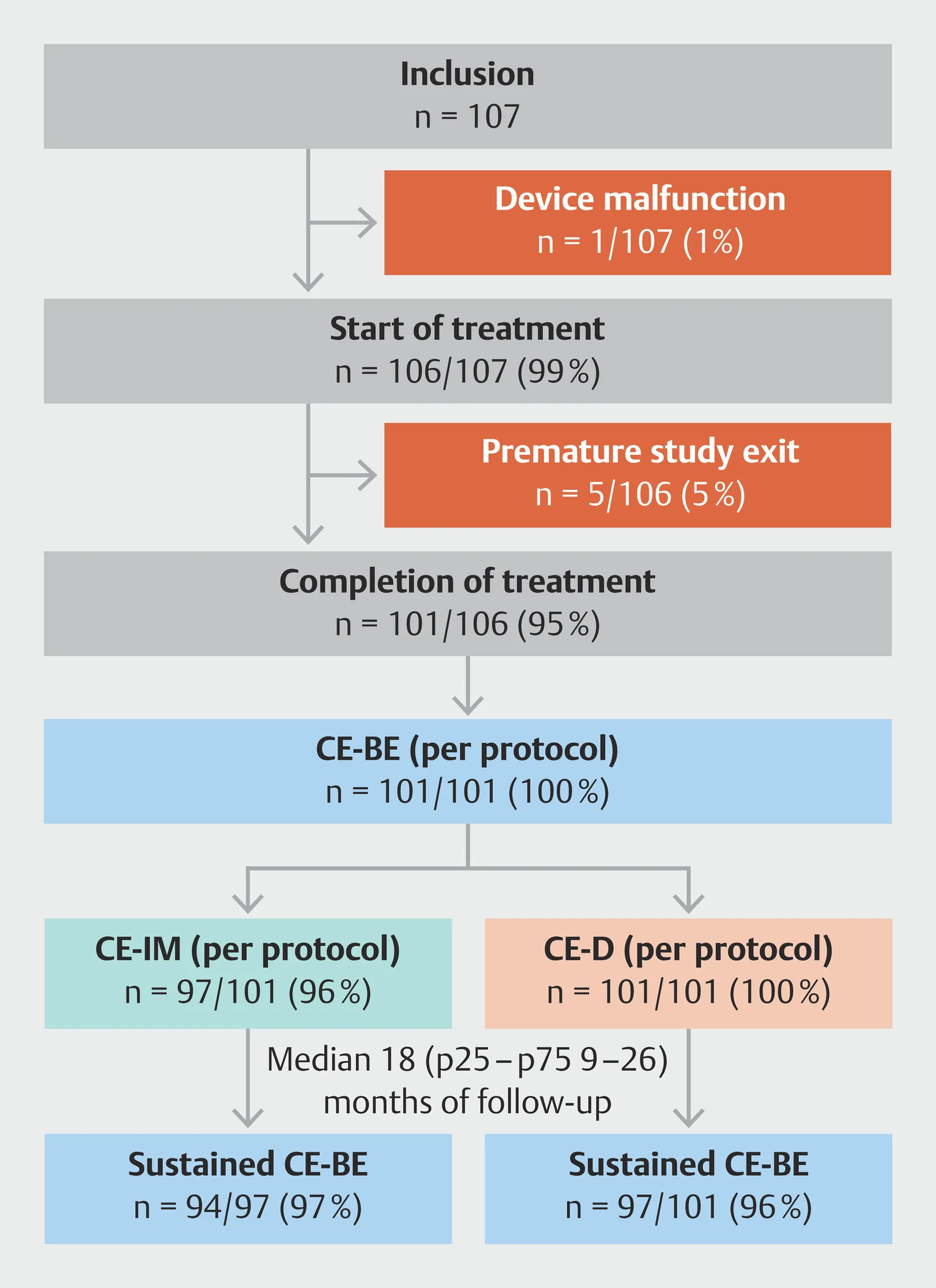
Flow chart of patient inclusion and treatment outcomes. One of the 107 included patients could not be treated with focal cryoballoon ablation as a result of a device malfunction, requiring a switch to radiofrequency ablation, and five patients discontinued treatment, resulting in 101 patients who finished the treatment phase per protocol. BE, Barrett esophagus; CE, complete eradication; D, dysplasia; IM, intestinal metaplasia.

After each FCBA treatment, the endoscopist was asked to record a score reflecting overall procedural satisfaction, using a numeric rating scale from 1 (poor) to 10 (excellent). Furthermore, patients were asked to register retrosternal pain at rest and during eating (numeric rating scale from 0 [no pain] to 10 [worst pain ever experienced]), dysphagia (validated scale from 0 [no dysphagia] to 4 [no passage even for liquids]), and analgesic use in a daily symptom diary over 14 days ( **Fig. 2s** ). In addition, patients were contacted on Day 7 (±2 days) to evaluate retrosternal pain, dysphagia, analgesic use, time to resume normal daily activities, and adverse events.

After completion of the treatment phase, patients entered follow-up, consisting of endoscopic surveillance at 6 months and annually thereafter up to 5 years. During follow-up, biopsies of any recurrent BE were taken at the discretion of the local principal investigator(s). Given that the predefined 5-year follow-up period is still ongoing, we present here the interim follow-up results to date.

### Outcomes

The primary outcomes were as follows.

1. Complete eradication of BE (CE-BE), defined as absence of any endoscopically visible Barrett mucosa after completion of the treatment phase.
2. Complete eradication of intestinal metaplasia (CE-IM), defined as absence of IM in all biopsies obtained at the first endoscopy with CE-BE or after the final FCBA of the GEJ for persisting IM.
3. Complete eradication of dysplasia (CE-D), defined as absence of LGD, HGD, and cancer in all biopsies obtained at the first endoscopy with CE-BE, after the final FCBA of the GEJ for persisting IM, or from residual BE after the maximum number of FCBA and add-on treatment sessions.

The secondary outcomes were as follows.

1. Adverse events, defined as acute (during the procedure), early (0–48 hours after the procedure), and late (>48 hours after the procedure). Adverse events were graded I to V according to the definitions of the AGREE classification 18 . In cases of esophageal stricture, endoscopic dilation was typically repeated at a 2-week interval until resolution of dysphagia. A stricture was considered severe when >3 endoscopic dilations were required.
2. Feasibility and tolerability, assessed through several outcome variables as defined in **Table 2s** . For the tolerability analyses, we evaluated the symptom diaries completed after the first FCBA session, given that the symptom diaries of consecutive sessions were potentially influenced by the patient’s habituation to pain and decreasing BE length. In cases with a missing symptom diary, reported scores during the follow-up phone call at Day 7 were included in the analyses.
3. Durability of the treatment response during follow-up, with follow-up defined as the interval between the first post-treatment control endoscopy with no endoscopically visible BE and the last follow-up endoscopy or date of recurrence.

### Histopathological evaluation

Histopathological assessments at baseline and during treatment were performed by the local expert pathologist at each study site. ER specimens were evaluated for dysplasia and cancer including tumor invasion depth, differentiation grade, presence of lymphovascular invasion, and radicality at the vertical resection margins. Biopsies were evaluated for presence of IM or dysplasia, and additionally for the presence of buried Barrett glands in those from the neosquamous epithelium.

### Statistical analysis

The sample size calculation was based on the primary efficacy outcome. A sample size of 97 was required for a 95%CI with a 6% margin of error when CE-IM was expected to be 90%. The reference CE-IM rate was based on prior studies on FCBA (CE-IM 88%) 14 and RFA (CE-IM 88%–93%) 4 5 19 . Assuming a 10% dropout rate, the targeted enrollment was 107.

Statistical analysis was performed using the Statistical Software Package SPSS version 27 for Windows (IBM Corp., Armonk, New York, USA). For descriptive statistics, mean with SD and median with 25th and 75th percentiles (p25–p75) were used for variables with respectively normal and skewed distributions. Outcome variables were reported as proportions or medians, including 95%CIs where appropriate. All reported 95%CIs were constructed using simple bootstrapping with 1000 samples. For esophageal stricture formation, an additional univariable logistic regression analysis was performed to identify potential risk factors (i.e. BE length, prior ER, and median number of ablations per FCBA session). Tolerability and durability analyses were performed in R version 4.3.2 for Mac (R Foundation for Statistical Computing, Vienna, Austria). Durability was estimated using both crude proportions and the Kaplan–Meier method. All analyses were considered exploratory, and no formal correction for multiple testing was applied.

### Ethics

The study protocol and subsequent amendments were reviewed and approved by the Medical Research Ethics Committees United (NL64555.100.18). In addition, the institutional review board of each participating center approved the study protocol. Written informed consent was obtained from all patients participating in the study. An independent data and safety monitoring board was established to monitor patient safety and to decide on continuation of the study based on interim analyses conducted after enrollment of 25% and 50% of the planned sample size.

## Results

### Study population

Between November 2019 and July 2022, 107 patients were included. Baseline characteristics are summarized in Table 1 . The mean age of patients was 65 years (SD 10), and the majority were male (91/107; 85%). The median pre-ablation BE length was C0M2 (p25–p75 C0–1M1–3), and contained LGD (32%), HGD (32%), or early cancer (37%) as worst baseline histopathology. ER at entry was performed in 69/107 patients (65%).

**Table:** **Table 1** Baseline characteristics.

	Intention to treat (n = 107)	Per protocol (n = 101)
Male sex, n (%)	91 (85)	85 (84)
Age, mean (SD), years	65 (10)	65 (10)
BMI, mean (SD), kg/m ^2^	28 (5)	27 (5)
ASA classification, n (%)		
ASA I	23 (22)	22 (22)
ASA II	69 (65)	65 (64)
ASA III	15 (14)	14 (14)
ASA IV	0 (0)	0 (0)
Prior fundoplication, n (%)	3 (3)	3 (3)
Hiatal hernia, n (%)	98 (92)	92 (91)
Hiatal hernia length, mean (SD), cm	2 (1)	2 (2)
Prior endoscopic resection, n (%)	69 (65)	65 (64)
Endoscopic resection technique, n (%)		
EMR-Cap	19 (18)	15 (15)
EMR-MBM	45 (42)	45 (45)
ESD	5 (5)	5 (5)
Endoscopic resection type, n (%)		
En bloc	33 (31)	30 (30)
Piecemeal ^1^	36 (34)	35 (35)
Worst pre-ablation histology, n (%)		
LGD	34 (32)	30 (30)
HGD	34 (32)	34 (34)
Mucosal cancer	36 (34)	35 (35)
Submucosal cancer	3 (3)	2 (2)
Pre-ablation BE extent, median (p25–p75), cm		
Circumferential	0 (0–1)	0 (0–1)
Maximum	2 (1–3)	2 (1–3)
BE, Barrett esophagus; BMI, body mass index; EMR, endoscopic mucosal resection; EMR-Cap, cap-assisted EMR; EMR-MBM, EMR with multi-band mucosectomy device; ESD, endoscopic submucosal dissection; HGD, high grade dysplasia; LGD, low grade dysplasia; p25–p75, 25th to 75th percentile. ^1^ Median number of resected pieces was 2 (p25–p75 2–4).		

As can been seen in Fig. 2 detailing the flow of patients throughout the study, one patient (1/107; 1%) was not treated with FCBA owing to a device malfunction requiring a switch to RFA. In addition, five patients (5/107; 5%) left the study prematurely after a median of 2 FCBA sessions (min–max 2–4) due to a withdrawal of consent (n = 1), an investigator’s decision due to recurrent esophagitis as a result of the patient’s inability to take high-dose proton pump inhibitors (n = 1), or unrelated comorbidity (n = 3). The remaining patients (101/107; 94%) finished the treatment phase per protocol.

### Feasibility

As shown in Table 2 , patients underwent a median of 2 FCBA sessions (p25–p75 2–3). The majority of patients (97/107; 91%) underwent at least two FCBA sessions according to the protocol. In the remaining patients, a second FCBA was omitted due to a premature study exit (n = 1), protocol deviation (n = 4), or scarring resulting in the physician’s decision to refrain from a second ablation owing to fear of (recurrent) stricture formation (n = 5). Throughout the study, a total of 248 FCBA procedures were performed, during which a device malfunction occurred in 8% (20/248; 95%CI 5%–12%). One device malfunction required a switch to RFA as a result of an ongoing controller error, while in all other cases with device malfunctions, the FCBA procedure could be completed successfully after replacement of an FCBA component.

**Table:** **Table 2** Overview of treatment characteristics.

	Intention to treat (n = 107)	Per protocol (n = 101)
No. of FCBA sessions, median (p25–p75)	2 (2–3)	2 (2–3)
Additional FCBA for persisting IM at endoscopically normal GEJ, n (%)	7 (7)	7 (7)
Procedure time per FCBA session, median (p25–p75), minutes	18 (13–24)	18 (13–23)
Ablation time per FCBA session, median (p25–p75), minutes	9 (6–12)	9 (6–13)
No. of ablations per FCBA session, median (p25–p75)	7 (5–9)	7 (5–9)
Add-on treatment, n (%)		
APC	41 (38)	41 (41)
EMR	2 (2)	2 (2)
Total no. of treatment sessions, median (p25–p75)	3 (3–4)	3 (2–3)
APC, argon plasma coagulation; EMR, endoscopic mucosal resection; FCBA, focal cryoballoon ablation; GEJ, gastroesophageal junction; IM, intestinal metaplasia; p25–p75, 25th to 75th percentile.		

The median procedure and ablation times were 18 minutes (p25–p75 13–24) and 9 minutes (p25–p75 6–12), respectively. A median of 7 ablations (p25–p75 5–9) were performed per FCBA session ( Table 2 ).

The technical success rate was high, with all visible BE treated as intended in 242/248 (98%; 95%CI 96%–99%) FCBA procedures. The feasibility of FCBA was further reflected by a high satisfaction score (median 8 [p25–p75 7–9]) recorded by the treating endoscopists.

### Add-on treatment

After at least two FCBA sessions, add-on treatment was performed in 43/107 patients (40%; 95%CI 32%–50%), most of whom were treated with APC for small remaining nonsuspicious islands (41/107; 38%; 95%CI 30%–48%) with a median of 1 session (p25–p75 1–1). Only two patients underwent an add-on EMR for residual Barrett mucosa ( Table 2 ). In one of these cases, the treating endoscopist decided to resect instead of ablate an island of 1 cm near the GEJ after the patient suffered a severe stricture requiring four dilations immediately following the first FCBA session ( **Table 3s** ). The other patient had a BE island of 1 cm in the tubular esophagus 5 cm above the GEJ, which was resected en bloc with EMR due to an irregular aspect, although pathology showed no dysplasia. Overall, the median total number of treatment sessions (i.e. FCBA and add-on therapy) was 3 (p25–p75 3–4) ( Table 2 ) with a median treatment period of 8 months (p25–p75 6–10).

### Efficacy

All endoscopically visible BE was eradicated in 101/107 patients (94%; 95%CI 90%–98%) and 101/101 patients (100%; 95%CI 100%–100%) by intention-to-treat and per-protocol analyses, respectively. Biopsies from the GEJ after CE-BE showed persisting IM in 8/107 patients (8%; 95%CI 3%–13%). In the majority of these patients (7/8; 88%), one additional FCBA of the GEJ was performed according to the protocol ( Table 2 ). Ultimately, CE-IM was reached in 91% (97/107; 95%CI 85%–95%) and CE-D in 94% (101/107; 95%CI 90%-98%) by intention-to-treat analysis. In the per-protocol analysis, CE-IM and CE-D were achieved in 96% (97/101; 95%CI 92%–99%) and 100% (101/101; 95%CI 100%–100%), respectively ( Fig. 2 ).

While CE-D was achieved in all patients who completed the study per protocol, CE-IM was not achieved in four patients. Two patients had persisting IM at the GEJ after one final additional FCBA of the GEJ, whereas the other two patients had persisting IM at the GEJ after an elective decision to withhold further treatment due to unrelated comorbidity (n = 1) or for prevention of recurrent stricture formation (n = 1). None of the patients required additional off-protocol endoscopic treatment after completing the maximum number of FCBA and add-on treatment sessions.

In the biopsies obtained from the neosquamous epithelium, buried BE glands were detected in 1/107 patients (1%; 95%CI 0%–3%) in only a single biopsy. This patient was treated successfully for a C2M5 BE after a prior EMR for a low-risk, well differentiated, T1m3 cancer. As no endoscopically visible Barrett mucosa or buried BE glands were detected during two follow-up endoscopies at 6 and 18 months, no additional endoscopic treatment has been performed thus far.

### Disease progression

During the treatment phase, 3/107 patients (3%; 95%CI 0%–7%) developed a new, visible, neoplastic lesion that was amenable to ER. One of these patients had received a single FCBA session for a C2M3 flat BE containing LGD before a Paris 0-IIa lesion of 2 cm was detected at the GEJ. After subsequent resection by ESD, histopathology showed HGD. In the second patient, a new Paris 0-IIb/c lesion of 1 cm was detected at the GEJ after a single FCBA session for a C0M1 BE originally containing a low-risk T1m3 cancer removed by EMR. This new pop-up lesion containing HGD was resected through ESD. The last patient with progression developed a lesion of 2.5 cm at the GEJ classified as Paris 0-Is-IIa after three prior FCBA sessions for C0M1 flat BE with HGD. After resection through ESD, histopathology revealed a moderately differentiated T1m3 cancer with no lymphovascular invasion. Eventually, all three patients achieved CE-IM after a median of 2 FCBA sessions (min–max 1–3).

### Safety

No acute or early adverse events occurred during any of the study procedures. Esophageal stricture was the most common adverse event in 13/107 patients (12%; 95%CI 7%–19%; per-protocol analysis 13/101 [13%]; 95%CI 6%–20%), which resolved after a median of 2 dilations (p25–p75 1–3). While 3/107 patients (3%; 95%CI 0%–7%) developed a severe stricture requiring >3 dilations (4, 5, and 6 dilations, respectively), none of the patients with a stricture required stent placement or incisional therapy ( **Table 4s** ). In general, strictures developed after a median of 1 FCBA session (p25–p75 1–1) and within a median of 26 days (p25–p75 18–33) after the respective FCBA session. The details of all patients who developed a stricture are shown in **Table 3s** . An additional univariable logistic regression analysis identified the median number of ablations during each FCBA session per patient as an independent risk factor for stricture development (odds ratio 1.20; 95%CI 1.04–1.39) ( **Table 5s** ).

Five patients were admitted to hospital due to an adverse event not associated with FCBA, and three patients (3/107; 3%) died due to unrelated severe comorbidity ( **Table 4s** ).

### Tolerability

**Fig. 3:**
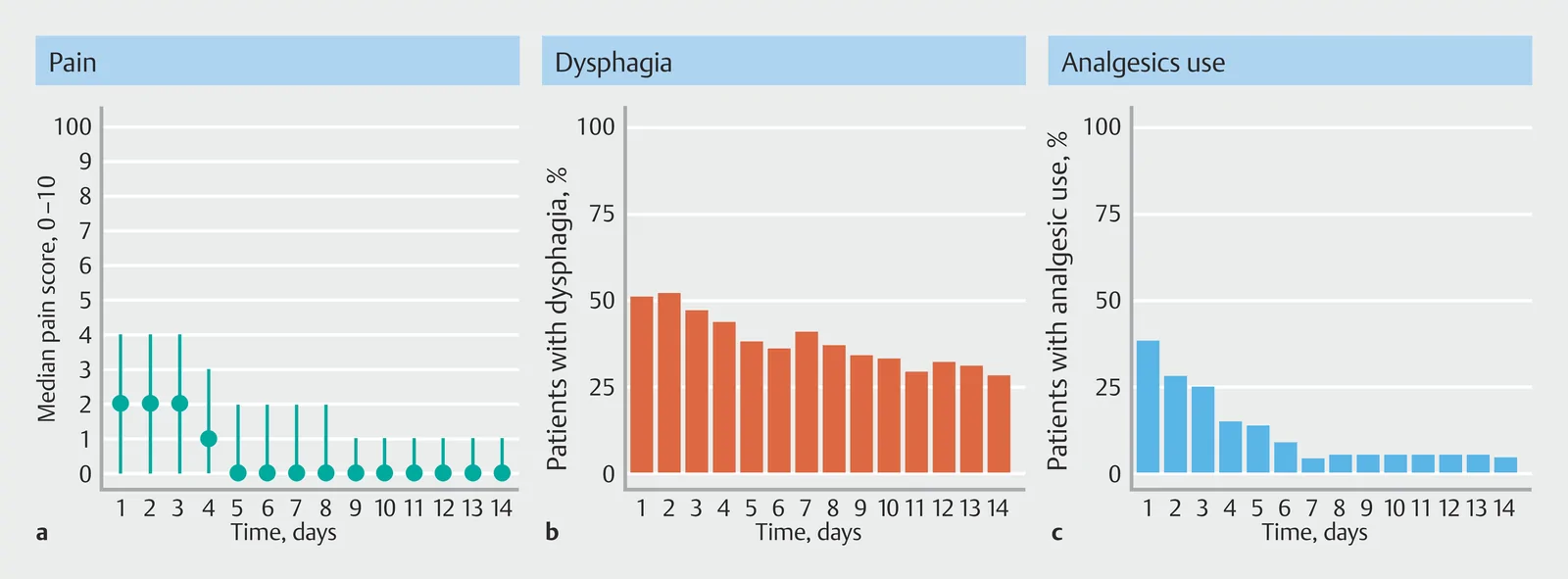
Tolerability during the 14-day period after focal cryoballoon ablation treatment. **a** Median composite pain score including the interquartile ranges indicated by the error bars. **b** Presence of dysphagia. **c** Use of painkillers. The composite pain score was defined as the maximum value of two questions in the symptom diary regarding pain at rest and pain when eating (scale 0–10, with 0 indicating no pain and 10 indicating worst pain ever experienced).

Six symptom diaries (6%) were missing for the 107 first FCBA sessions. Based on all available symptom diaries, FCBA was well tolerated with low post-procedural pain scores and low incidence of dysphagia and analgesic use ( Fig. 3 ). Overall, 47/107 patients (44%) reported having to adjust their daily activities after treatment with FCBA, with normal daily activities generally being resumed in a median of 2 days (p25–p75 1–3).

### Durability of treatment response

Of the 101 patients who entered follow-up after CE-D was established, 96% (97/101; 95%CI 92%–99%) remained free of endoscopically visible Barrett epithelium. The majority of patients with a recurrence (3/4; 75%) only had small visible islands, which were retreated with APC (n = 2) or kept under endoscopic surveillance (n = 1). The remaining patient was diagnosed with a recurrent Barrett tongue of C0M1 at 18 months post-treatment, which was re-treated with FCBA. More importantly, none of the patients who entered follow-up developed a neoplastic recurrence.

Of the 97 patients who achieved CE-IM after treatment, CE-BE was maintained in 97% (94/97; 95%CI 92%–100%). The majority with a recurrence (2/3; 67%) only had small visible islands, and the remaining patient was diagnosed with a nondysplastic Barrett tongue, as described above.

Notably, besides the aforementioned patients with recurrences, 12 patients (12/101; 12%) were diagnosed with small BE islands during the first follow-up endoscopy after CE-IM or CE-D was established. These patients were not considered as recurrent BE but were assumed to have persistent small BE islands overlooked at the first follow-up endoscopy. While three of these patients were kept under endoscopic surveillance, the majority of patients were successfully re-treated with APC (n = 9).

**Fig. 4:**
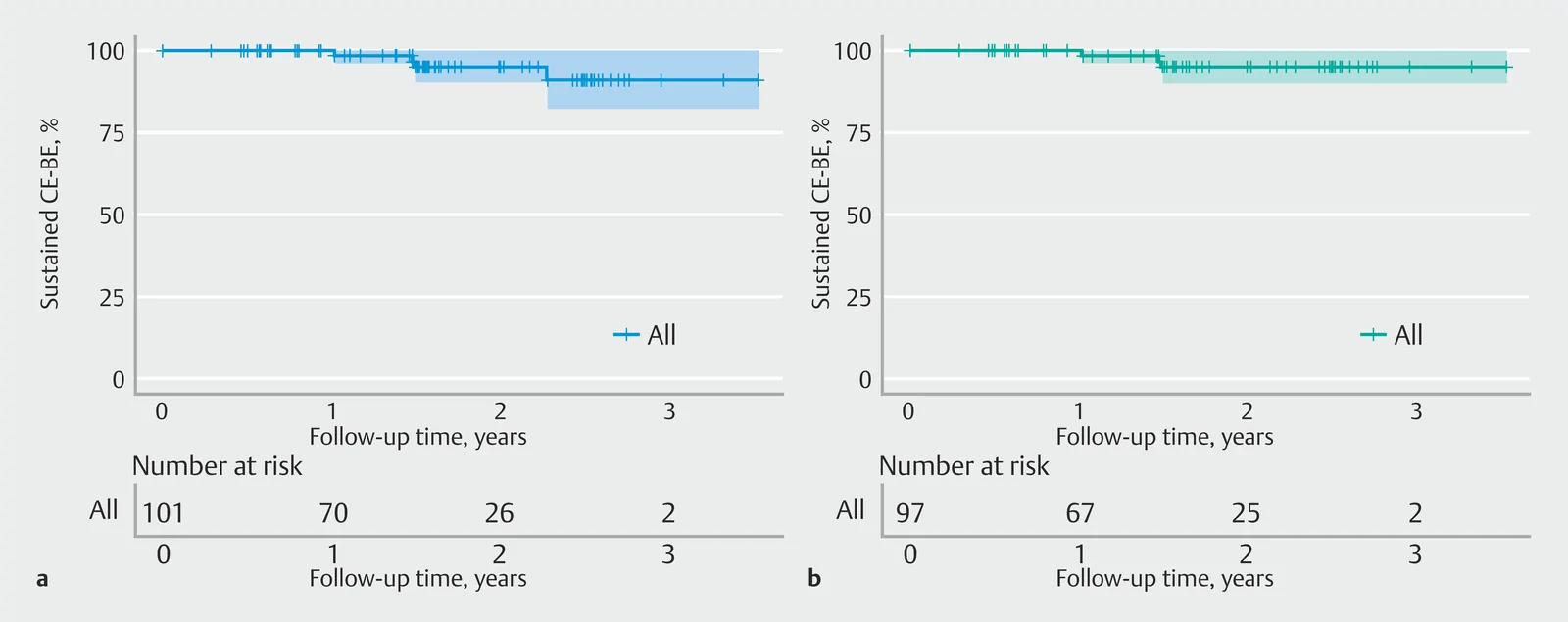
Durability of treatment response after focal cryoballoon ablation treatment. This figure depicts the Kaplan–Meier curves showing the estimated cumulative incidence rates of sustained complete eradication of Barrett epithelium (CE-BE) during follow-up, after complete eradication of dysplasia ( **a** ) and intestinal metaplasia ( **b** ) was established in 101 and 97 patients, respectively. Any recurrent visible Barrett epithelium after the first post-treatment control endoscopy with no endoscopically visible BE was considered a failure for recurrence-free survival, even though complete eradication was re-established after additional endoscopic treatment. Patients who discontinued follow-up were censored at the date of their last control endoscopy.

Fig. 4 shows the Kaplan–Meier curves for the cumulative rates of sustained CE-BE after the first negative control endoscopy for the patients who achieved CE-D (n = 101) and CE-IM (n = 97). During a median follow-up of 18 months (p25–p75 9–26; min–max 0–42), patients underwent a median of 2 follow-up endoscopies (min–max 0–4).

## Discussion

This is the largest prospective, multicenter study to date on endoscopic eradication therapy with multifocal cryoballoon ablation for BE of limited extent with early neoplasia using a therapeutic dose of 8 seconds per ablation. This trial confirms that FCBA is safe, well tolerated, and effective for the induction and sustainment of complete eradication. Treatment typically consisted of two repetitive FCBA sessions complemented with APC for minute BE islands. Also, additional ER with ESD proved to be a safe rescue modality for diagnosis and treatment of new visible lesions during the treatment phase.

The ease of use of FCBA was reflected in the high technical success rate and high satisfaction scores reported by the treating endoscopists. Furthermore, ablation and procedure times were relatively short (9 and 18 minutes, respectively), especially compared with other studies on FCBA (11–17 and 30 minutes, respectively) 14 15 . Nonetheless, the incidence of device malfunctions was substantial (8%); most device malfunctions could be resolved easily by replacing one of the FCBA components, but one case required a switch to RFA. More importantly, endoscopic eradication therapy with FCBA was shown to be highly effective, with high eradication rates for dysplasia (intention to treat 94%; per protocol 100%) and IM (intention to treat 91%; per protocol 96%). Although these eradication rates are higher than those reported in similar studies on RFA, which demonstrated CE-D and CE-IM in 81%–93% and 77%–88%, respectively 4 5 6 , it should be noted that our study consisted of a more favorable subset of patients with smaller BE segments (median C0M2). A similar rationale applies to hybrid APC, another alternative heat-based technique with reported CE-IM rates ranging from 79% to 100% 20 21 22 . While the observed efficacy of FCBA appears comparable, hybrid APC was generally applied in patients with longer BE segments, which may indicate a higher therapeutic potential.

Notably, our eradication rates lie at the upper end of the spectrum compared with other recent similar cohort studies on FCBA, which reported CE-D and CE-IM rates of 76%–95% and 72%–88%, respectively 14 15 . Our high rates of complete eradication may be partly attributed to the rigorous treatment of the GEJ, which was ablated circumferentially at least twice in nearly all patients, irrespective of any remaining BE. Another important difference from other trials on FCBA is the strict quality control, which involved exclusive participation of expert centers, hands-on training for each of the treating endoscopists, and on-site supervision during each treatment endoscopy to ensure adherence to the treatment protocol.

Once CE-BE, CE-D, and CE-IM was established, CE-BE was maintained in the majority of patients (96%, 96%, and 97%, respectively). Despite these high rates of sustained eradication, it must be noted that follow-up is still ongoing and complete durability analysis will be reported after 5 years of follow-up. Thus far, most recurrences consisted of small BE islands, whereas only a single patient was diagnosed with a recurrent flat Barrett tongue. More importantly, all recurrences were managed endoscopically, and no neoplastic recurrences were detected during follow-up. In addition to these “actual” recurrences, a small group of patients were found to have small islands during the first control endoscopy at 6 months post-treatment. These findings imply that follow-up may have been initiated too early, despite negative biopsies, as these small islands were likely overlooked and not all endoscopically visible BE was completely eradicated at the first follow-up endoscopy; diminutive islands can easily be missed. However, whether these small islands represent residual BE that was initially missed or true recurrences cannot be determined with absolute certainty. Compared with a previous study on FCBA, APC had to be used more frequently to patch up small BE islands (3% vs. 40%, respectively) 15 . Nevertheless, APC is a cheap and widespread endoscopic technique that can be used complementarily to ER and any type of ablation method, especially considering that endoscopic eradication therapy is a multimodal treatment strategy. Therefore, the use of APC was not restricted in our study, in contrast to the study from Canto et al. where APC could only be applied in cases with <3 remaining BE islands 15 . However, the application of APC was also lower in a comparable landmark study on RFA (11%) 5 . This difference might be a direct consequence of small untreated areas that arise when overlapping ablations are avoided during FCBA, resulting in a higher probability of residual BE islands.

As for safety, our stricture rate of 13% is within the range of other studies, which report strictures in 10%–16% after treatment with FCBA 14 15 , and is, as expected, lower compared with our separate cohort that was treated with the initial dose of 10 seconds (21%; **Supplementary Text 1s** ). Compared with similar landmark studies on RFA, reporting stricture rates of 7%–12% and including patients with relatively longer BE segments (median ≤6 cm) 4 5 6 , the risk of stricture seems to be somewhat higher after FCBA. Similarly, hybrid APC has shown even lower reported stricture rates of 0%–4% 20 21 22 , despite being used in patients with longer BE segments, further suggesting a potentially lower risk profile than FCBA. One potential contributing factor to the risk of stricture formation after FCBA is the overtreatment of specific areas as a result of overlapping ablations. This theory is supported by the fact that the median number of ablations per FCBA session was an independent risk factor for stricture formation. In addition, 6/13 patients (46%) who developed a stricture received more than 10 ablations per FCBA session, which is higher than the median number of 7 ablations ( **Table 3s** ). These findings emphasize the paramount importance of carefully preventing any overlap of ablations or reducing the dose to 6 seconds when overlapping is inevitable. Based on these observations, in combination with the established high efficacy rates when using 8 seconds as the standard dose, 6 seconds might be an interesting dose for future research to further improve the safety profile of cryoballoon ablation.

Our findings may support the notion that FCBA offers improved tolerability compared with RFA, a potential advantage that has been suggested in the literature 10 . In our cohort, dysphagia was reported in 52% of patients ( Fig. 3 ), which is lower than the 83% reported in a recent observational study of patients treated with RFA 8 . Additionally, the median post-procedural pain score following FCBA did not exceed 2 ( Fig. 3 ), which is consistent with earlier reports on the tolerability of FCBA 10 14 15 16 . Given that a pain score above 5 was reported in 43% of RFA procedures 8 , these findings may point toward a more favorable post-procedural pain profile for FCBA. However, any suggestion of improved tolerability with FCBA should be interpreted with caution, as no direct head-to-head comparison was performed, and our cohort consisted of patients with BE segments of limited length, which may have influenced outcomes such as dysphagia and pain.

One of the strengths of this study is the large sample size with low dropout rate, resulting in a high number of patients in the per-protocol analysis. Another important strength is the European multicenter setting in Barrett expert centers, which enabled a study population with widespread demographic background. Moreover, the standardized treatment and follow-up protocol was largely based on the current recommendations for endoscopic eradication therapy and therefore mimics daily clinical practice. All FCBA procedures were performed solely by trained and experienced endoscopists to ensure standardization of the technique. In addition, high-quality data and adherence to the protocol were guaranteed by attendance of a study representative on site at almost every FCBA session, even during the start of the COVID-19 pandemic in 2020. Finally, we were able to evaluate post-procedural tolerability through patient-reported outcomes, which may be relevant for future guidance and counseling of patients in the choice for ablation technique.

This study also has several limitations that need to be addressed. First, this prospective trial was designed as an interventional study with no comparative arm. The lack of standardization of the number of ablations and the decision to further reduce the dose to 6 seconds left room for interuser variability, even though the treating endoscopists were extensively trained in the technique. Although multiple centers were involved, we did not adjust for center effects in our analysis. This was a deliberate choice given the relatively small sample size and our substantial efforts to minimize the intercenter variability through investigator meetings, endoscopist training, and close treatment monitoring. Another limitation that should be noted is that the results of this study may not be applicable to patients with longer Barrett segments; we only treated BE of limited extent (maximum C2M5) owing to the focal nature of the current device. The generalizability of our results is also limited by the exclusion of patients who required a more extensive ER, which is associated with a higher risk of stricture formation 7 . Moreover, the results of this study cannot automatically be extrapolated to general clinical practice, as all patients underwent endoscopic work-up and treatment in dedicated centers with expertise in BE care. However, current guidelines strongly recommend the centralization of endoscopic management of patients with Barrett neoplasia in expert centers that meet essential criteria, such as sufficient experience in ER and ablation techniques, expert pathology review, adequate annual case load, case discussion in multidisciplinary meetings, and access to esophageal surgery 2 3 . The expert setting of this study also led to the omission of routine histological sampling during follow-up after successful treatment. Although this may have resulted in an underestimation of the recurrence rates, the clinical significance of IM in an endoscopically normal esophagus remains questionable 23 24 . Finally, some therapeutic sessions had to be postponed due to a lack of endoscopy capacity during the COVID-19 pandemic, which may have negatively influenced the total treatment time per protocol.

Given that a versatile ablation tool must be able to treat BE of different sizes, several essential steps need to be taken before cryoballoon ablation will find more widespread implementation in future clinical practice. A prior study from our research group has evaluated a cryoballoon ablation device that enables treatment of larger surface areas 17 . Despite treatment with this large-area device appearing to be feasible, the exact dosing regimen is currently still under investigation in a dose de-escalation study (NCT05740189). In the future, the combination of initial treatment with this large-area device followed by treatment with the focal device for complete eradication should be investigated in patients with longer BE segments. Finally, direct head-to-head comparison between cryoballoon ablation and RFA is lacking, although the two techniques should not necessarily need to compete and might be complementary modalities in the future.

To conclude, in expert hands, endoscopic eradication therapy with multifocal cryoballoon ablation demonstrated high efficacy for the treatment of BE of limited extent, although the risk for stricture formation warrants consideration. Our results should be interpreted with caution given the absence of a direct comparator and the strictly selected cohort limiting external validity.

## Supplementary Material

Supplementary Material Zusatzmaterial
