# Supplementary material for: Endoscopic eradication therapy with multifocal cryoballoon ablation for Barrett esophagus-related neoplasia: a prospective European multicenter study
Source: Endoscopy. 2025 Nov 19;58(4):323–33. doi: 10.1055/a-2710-6551 (PMC13494755; doi:10.1055/a-2710-6551)
Supplement: Supplementary file 1 [file 10-1055-a-2710-6551_27312299.pdf]

SUPPLEMENTARY MATERIAL

Endoscopic eradication therapy with multifocal cryoballoon ablation for Barrett esophagus-related neoplasia: a prospective European multicenter study

Charlotte N. Frederiks, Anouk Overwater, Torsten Beyna, Horst Neuhaus, Raf Bisschops, Roos E. Pouw, Jacques J. G. H. M. Bergman, Maximilien Barret, Roberta Maselli, Vinay Sehgal, Rehan J. Haidry, Bas L. A. M. Weusten

Supplementary Text 1. Cohort treated with the initial dose of 10 seconds per ablation.

Amendment of initial dose

The initial starting dose of this prospective multicenter study was based on previous studies, which established 10 seconds as standard dose for focal cryoballoon ablation (FCBA). [12-14] Nevertheless, after enrollment of the first 28 of the planned 107 patients, the stricture rate was higher than expected based on previous literature, which led to a temporary pause of the study. This difference can be explained by the fact that the first-generation device was used in the earlier studies, while for the current study the next-generation device became available. This next-generation system was improved by adding a foot pedal and expanding the size of the cartridges, resulting in more energy delivery and ice formation. Eventually, an extensive technical analysis revealed that a dose of 8 seconds for the next-generation device was comparable to a dose of 10 seconds with the first-generation device, after which the study was amended to restart with a dose of 8 seconds per ablation. [16] As a result, the patients who received their initial treatment with a dose of 10 seconds were subsequently treated with 8 seconds during consecutive FCBA sessions until complete eradication of Barrett esophagus (BE). Hereafter, we report the most important outcomes for this separate cohort of 28 patients.

10 seconds cohort

Between April 2019 and October 2019, 28 patients were included in the cohort that was initially treated with 10 seconds. The majority was male (26/28; 93%) with a mean age of 66 years (SD 11) and a median BE length of C0M2 (p25-p75 C0-0M1-3). Early mucosal cancer was the most common worst baseline histology (12/28; 43%) followed by low grade dysplasia (8/28; 29%) and high grade dysplasia (8/28; 29%). Before entry, endoscopic resection was performed in 17/28 (61%) patients with endoscopic mucosal resection being the most used technique (15/17; 88%). While 27/28 (96%) patients finished the treatment phase per protocol, one (1/28; 4%) patient could not be treated with FCBA due to a device malfunction requiring a switch to radiofrequency ablation.

Patients underwent a median of 2 (p25-p75 2-3) FCBA sessions of which a median of 2 (p25-p75 1-2) procedures were performed with the initial dose of 10 seconds. While nearly half of the patients (13/28; 46%) completed the treatment phase with the 10-second dose, the remaining patients were treated

with the amended dose of 8 seconds during consecutive FCBA sessions. The majority of patients (25/28; 89%) underwent at least two FCBA sessions according to protocol. In the remaining three patients, a second FCBA was omitted due to a premature study exit as a result of a device malfunction (n = 1) or scarring resulting in the physician's decision to refrain from a second ablation because of the fear of recurrent stricture formation (n = 2). Per FCBA session, a median of 6 (p25-p75 5-7) ablations were performed. While none of the patients required an add-on endoscopic resection, 11/28 (39% [95% CI 21%-57%]) patients received add-on treatment with argon plasma coagulation for small remaining BE islands (median 1 [p25-p75 1-1] session). The total number of treatment sessions (i.e. FCBA and add-on therapy) was median 3 (p25-p75 2-3) with a median treatment period of 12 (p25-p75 6-14) months.

Efficacy

Complete eradication of all endoscopically visible BE (CE-BE) was achieved in 96% (27/28 [95% CI 89%-100%]) and 100% (27/27 [95% CI 100%-100%]) per intention-to-treat and per-protocol analysis, respectively. Biopsies from the gastroesophageal junction (GEJ) after CE-BE showed persisting IM in 3/28 (11% [95% CI 0%-21%]) patients who all received one final FCBA of the GEJ according to protocol. Eventually, complete eradication of intestinal metaplasia (CE-IM) and dysplasia (CE-D) was achieved in 26/28 (93% [95% CI 82%-100%]) and 27/28 (96% [95% CI 89%-100%]) by intention-to-treat analysis, respectively. In the per-protocol analysis, CE-IM and CE-D was reached in 26/27 (96% [95% CI 89%-100%]) and 27/27 (100% [95% CI 100%-100%]), respectively. One patient who completed the study per protocol failed CE-IM despite one final additional FCBA of the GEJ for persisting IM. None of the patients had buried BE glands in the biopsies from the neosquamous epithelium. One (1/28; 4% [95% CI 0%-11%]) patient progressed during the treatment phase. This patient had received two FCBA sessions for a C0M2 flat BE containing HGD when a Paris II-a lesion of 1 cm was removed at the GEJ by EMR. Histopathological evaluation of the endoscopic resection specimen revealed HGD, but subsequent biopsies from the GEJ confirmed the absence of dysplasia and IM.

Safety

No acute adverse events occurred during any of the study procedures. One patient experienced an early adverse event after FCBA consisting of an intravenous line-related thrombophlebitis, which resolved after antibiotics. Stricture requiring dilation was the most common adverse event in 6/28 (21% [95% CI 7%-39%]) patients (median 2 [p25-p75 1-4] dilations). Two (2/28; 7% [95% CI 0%-18%]) of these patients developed a severe stricture resolving after >3 dilations

(5 and 8, respectively). Strictures typically developed after a median of 1 (p25-p75 1-1) FCBA sessions and within a median of 26 (p25-p75 12-81) days after the respective FCBA session.

Durability of treatment response

During a median follow-up of 40 (p25-p75 31-42) months, patients underwent a median of 4 (p25-p75 3-4) follow-up endoscopies. Of the 27 patients who entered follow-up after CE-D was established, 85% (23/27 [95% CI 70%-96%]) remained free of endoscopically visible Barrett epithelium. Of the 26 patients who achieved CE-IM after treatment, CE-BE was maintained in 85% (22/26 [95% CI 68%-96%]). Two patients with a recurrence had small visible islands detected at 18 and 42 months post-treatment, while another patient was diagnosed with a recurrent Barrett tongue of C0M1 at 36 months post-treatment. All three patients were kept under endoscopic surveillance. The last patient with recurrence developed a neoplastic lesion at the GEJ 20 months post-treatment for which a curative EMR was performed with histopathology revealing HGD.

Table 1s Inclusion and exclusion criteria.

**Inclusion criteria**	**Exclusion criteria**
BE segment of C≤2 cm and M≤5 cm according to the Prague classification	Esophageal stricture preventing advancement of a therapeutic endoscope
Indication for ablation therapy as determined by:<br>1) Flat Barrett epithelium containing LGD or HGD, histopathologically confirmed by four quadrant biopsies of every 2 cm of the BE segment in the last 6 months prior to study participation;<br>2) Residual flat Barrett epithelium after endoscopic resection (either EMR or ESD) of a visible lesion at least 6 weeks prior to study participation, with histopathologic evaluation of the resection specimen revealing any degree of dysplasia or low-risk adenocarcinoma (i.e. invasion depth of <500 µm, good-to-moderate differentiation, absence of lymphovascular invasion, and tumor-negative resection margins[1])	Prior endoscopic resection >2 cm in length and/or >50% of the esophageal circumference
Ablation naïve (i.e. no previous ablation therapy of the esophagus)	Active reflux esophagitis, defined as Los Angeles grade B or higher
≥18 years of age at time of informed consent	Presence of esophageal varices
	History of achalasia
	Prior distal esophagectomy
	Severe medical comorbidities precluding endoscopy
	Uncontrolled coagulopathy
	Pregnant or planning to become pregnant during period of study participation
	Life expectancy ≤2 years, as judged by the local principal investigator

Abbreviations: BE, Barrett esophagus; C, circumferential extent; EMR, endoscopic mucosal resection; ESD, endoscopic submucosal dissection; HGD, high grade dysplasia; LGD, low grade dysplasia; M, maximum extent.
[1]After ESD both lateral as well as vertical resection margins had to be tumor-negative.

Table 2s Definition of feasibility and tolerability outcomes.

Outcomes	Definition
**Feasibility**	
Technical success	Treatment of all visible Barrett esophagus as intended.
Device malfunction	Technical failure of focal cryoballoon ablation system with the need for device replacement or switch to radiofrequency ablation.
Procedure time	Time in minutes between introduction and removal of the endoscope.
Ablation time	Time in minutes between insertion and removal of the endoscope and removal of the focal cryoballoon ablation catheter.
Satisfaction score	Score given by the endoscopist directly after the treatment session regarding the overall satisfaction on a scale of 1-10, with 1 indicating poor and 10 excellent.
**Tolerability**	
Composite pain score	Maximum value of two questions in the symptom diary regarding retrosternal pain in rest and when eating on a scale of 0-10, with 0 indicating no pain and 10 worst pain ever experienced.
Presence of dysphagia	Dichotomized value (present/absent) of the dysphagia score on a scale of 0-4, with 0 indicating no dysphagia and 4 no passage even for liquids.
Analgesic use	Dichotomized value (yes/no) of painkiller use.
Adjustment of normal daily activities	Dichotomized value (yes/no) of whether normal daily activities had to be adjusted after treatment.
Time to normal daily activities	Time in days until normal daily activities could be resumed after treatment.

Table 3s Details of patients who developed a stricture requiring dilation.

**Patient**	**Pre-ablation BE extent**, cm	**Prior ER**	**Axial length ER**, cm	**Circumferential extent ER**, %	**FCBA sessions**, n	**Ablations per FCBA session**, median n (min-max)	**Add-on treatment**	**Dilations**, n	**Treatment outcome**
1	C0M1	EMR	1	20	2	7 (5-8)	-	2	CE-IM
2	C0M1	EMR	1	42	1[1]	7 (-)	APC	1	CE-IM
3	C0M2	EMR	1	8	2	9 (8-9)	-	1	CE-IM
4	C0M2	EMR	1	10	2	7 (6-8)	APC	1	CE-IM
5	C0M2	EMR	1	25	3	7 (5-9)	-	1	CE-IM
6	C0M2	EMR	2	40	2	6 (5-7)	APC	1	CE-IM
7	C0M3	EMR	2	25	5	11 (9-17)	APC	3	CE-IM
8	C0M3	EMR	2	50	1[2]	12 (-)	EMR	4	CE-IM
9	C1M2	EMR	2	30	1[2]	20 (-)	-	2	CE-D
10	C1M4	EMR	2	50	2	10 (5-15)	APC	2	CE-IM
11	C2M3	-	-	-	2[3]	9 (2-16)	-	5	CE-IM
12	C2M3	EMR	1	20	1[2]	11 (-)	-	3	CE-IM
13	C2M5	EMR	2	25	2	14 (14-14)	APC	6	CE-IM

Abbreviations: APC, argon plasma coagulation; BE, Barrett esophagus; C, circumferential extent; CE-IM, complete eradication of intestinal metaplasia; CE-D, complete eradication of dysplasia; EMR, endoscopic mucosal resection; ER, endoscopic resection; ESD, endoscopic submucosal dissection; FCBA, focal cryoballoon ablation; M, maximum extent

[1]After a single FCBA a new, visible lesion was detected and subsequently resected by ESD.

[2]Treatment with a single FCBA as a result of a justified protocol deviation to prevent recurrent stricture formation.

[3]Treatment of gastroesophageal junction was omitted during the second FCBA session to prevent recurrent stricture formation.

Table 4s Overview of adverse events.

	Intention to treat (n = 107)	Severity
**Adverse events related to FCBA procedure**, n (%)		
Stricture requiring dilation[1]	13 (12)	Grade III (n = 13)
Poor healing[2]	11 (10)	Grade II (n = 10)
		Grade III (n = 1)
Food impaction	2 (2)	Grade III (n = 2)
Aspiration pneumonia	2 (2)	Grade II (n = 1)
		Grade IV (n = 1)
**Adverse events unrelated to FCBA procedure**, n (%)		
Heart disease	2 (2)	Grade III (n = 1)
		Grade V (n = 1)
Second primary tumor	2 (2)	Grade III (n = 2)
Fracture	1 (1)	Grade III (n = 1)
Infection	2 (2)	Grade II (n = 1)
		Grade V (n = 1)
Psychiatric disease	1 (1)	Grade V (n = 1)

[1]Stricture resolved after a median of 2 (p25-p75 1-3) dilations.

[2]Defined as active inflammatory changes with mucosal swelling, exudates and/or ulcerations observed during endoscopy ≥3 months post-FCBA.

Table 5s Univariable logistic regression analysis to identify potential risk factors for esophageal stricture formation.

**Variable**	**Odds ratio**	**95% CI**	**P value**
Pre-ablation circumferential BE extent	1.28	0.65-2.51	0.47
Pre-ablation maximum BE extent	1.15	0.75-1.76	0.51
Prior endoscopic resection	0.14	0.02-1.03	0.05
Median number of ablations per FCBA session	1.20	1.04-1.39	0.02
Abbreviations: BE, Barrett esophagus; CI, confidence interval.			

Fig. 1s The C2 Cryoballoon Ablation system (PENTAX Medical, Redwood City, California, USA) consisting of a controller with nitrous oxide cartridges stored under the black cap, foot pedal, and balloon catheter.

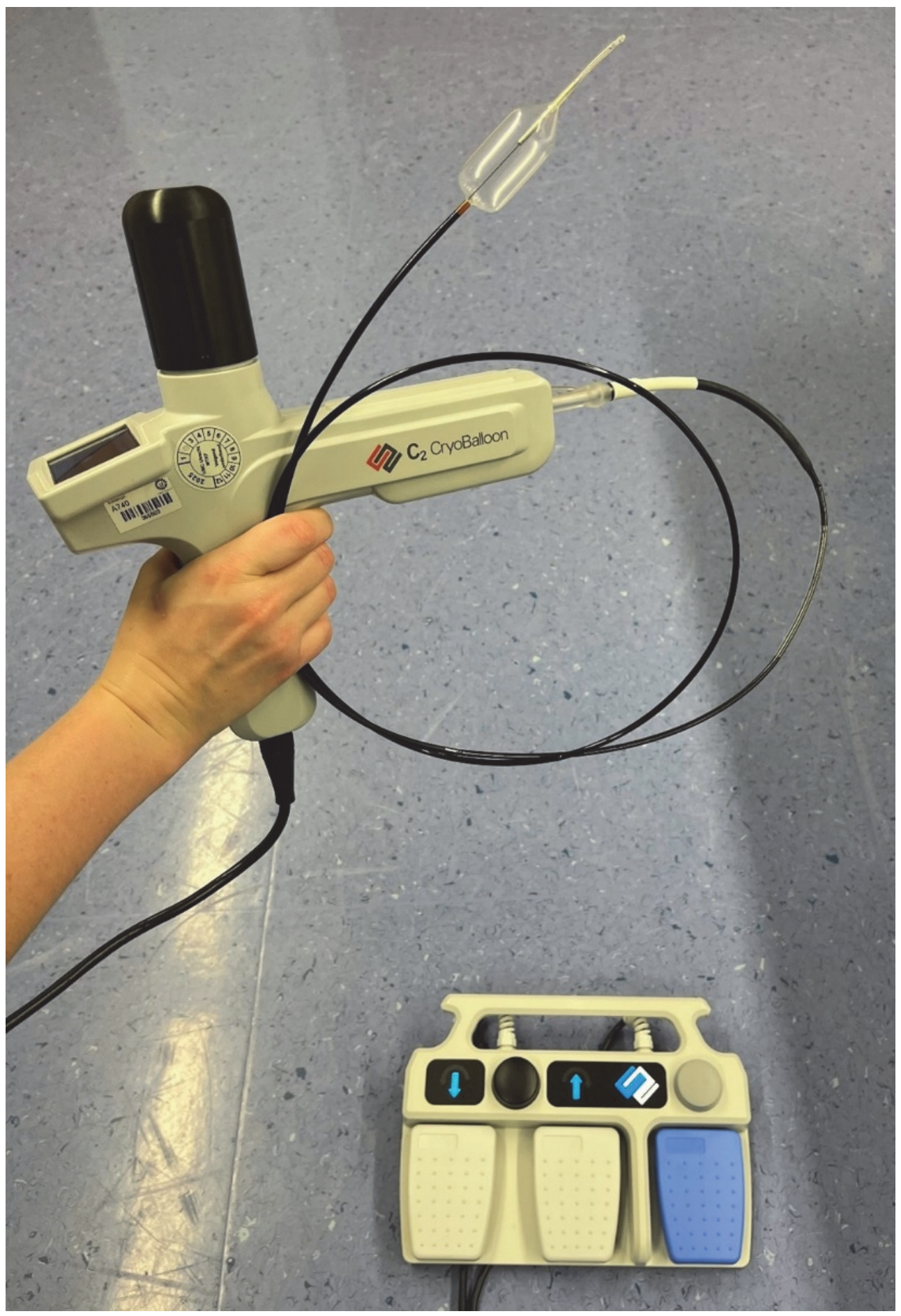

Fig. 2s Symptom diary to register retrosternal pain (both in rest and during meals), dysphagia, and analgesic use daily during 14 days after focal cryoballoon ablation treatment.

Day 1

The first two questions refer to PAIN after the treatment.

Please give a number between 0 and 10 which states the severity of pain experienced. A score of 0 indicates no pain at all. A score of 10 indicates the most severe pain you can imagine.

1. When you eat or drink, do you experience pain behind the breastbone? Note: You may only give one answer.	○	0	no pain at all
	○	1	
	○	2	
	○	3	
	○	4	
	○	5	
	○	6	
	○	7	
	○	8	
	○	9	
	○	10	severe and extreme pain
2. When you DO NOT eat or drink, do you experience pain behind the breastbone? Note: You may only give one answer.	○	0	no pain at all
	○	1	
	○	2	
	○	3	
	○	4	
	○	5	
	○	6	
	○	7	
	○	8	
	○	9	
	○	10	severe and extreme pain
3. Have you taken painkillers today? Note: you may give more than one answer.	□		No
	□		Yes, Paracetamol
	□		Yes, Ibuprofen, Brufen, Diclofenac or Voltaren
	□		Yes, another painkiller

The next question refers to how food and drinks pass down behind the breastbone.

4. How do you experience eating and drinking? Does it pass down easily? Note: you may only choose one answer. Choose the answer that best describes your symptoms.	○		No problem, I can eat and drink normally
	○		Some discomfort, but I can eat some solids
	○		Definite discomfort, I have to crush and puree all food
	○		A lot of discomfort, I can only drink, but not eat solids
	○		I cannot eat or drink
